# Understanding the Future Prospects of Synergizing Minimally Invasive Transforaminal Lumbar Interbody Fusion Surgery with Ceramics and Regenerative Cellular Therapies

**DOI:** 10.3390/ijms22073638

**Published:** 2021-03-31

**Authors:** Wen-Cheng Lo, Lung-Wen Tsai, Yi-Shan Yang, Ryan Wing Yuk Chan

**Affiliations:** 1 Department of Surgery, Division of Neurosurgery, School of Medicine, College of Medicine, Taipei Medical University, Taipei 11031, Taiwan; b101091096@tmu.edu.tw (Y.-S.Y.); b101095151@tmu.edu.tw (R.W.Y.C.); 2 Department of Neurosurgery, Taipei Medical University Hospital, Taipei 11031, Taiwan; 3 Taipei Neuroscience Institute, Taipei Medical University, Taipei 11031, Taiwan; 4 Department of Medical Education and Research, Taipei Medical University Hospital, Taipei 11031, Taiwan; lungwen@tmu.edu.tw

**Keywords:** MIS-TLIF, bone graft, ceramics, stem cells, platelet-derived biomaterials

## Abstract

Transforaminal lumber interbody fusion (TLIF) is the last resort to address the lumber degenerative disorders such as spondylolisthesis, causing lower back pain. The current surgical intervention for these abnormalities includes open TLIF. However, in recent years, minimally invasive TLIF (MIS-TLIF) has gained a high momentum, as it could minimize the risk of infection, blood loss, and post-operative complications pertaining to fusion surgery. Further advancement in visualizing and guiding techniques along with grafting cage and materials are continuously improving the safety and efficacy of MIS-TLIF. These assistive techniques are also playing a crucial role to increase and improve the learning curve of surgeons. However, achieving an appropriate output through TLIF still remains a challenge, which might be synergized through 3D-printing and tissue engineering-based regenerative therapy. Owing to their differentiation potential, biomaterials such as stem/progenitor cells may contribute to restructuring lost or damaged tissues during MIS-TLIF, and this therapeutic efficacy could be further supplemented by platelet-derived biomaterials, leading to improved clinical outcomes. Thus, based on the above-mentioned strategies, we have comprehensively summarized recent developments in MIS-TLIF and its possible combinatorial regenerative therapies for rapid and long-term relief.

## 1. Introduction

Spinal surgery is a final and effective resort to overcome the spinal degenerative disorders, spondylolisthesis, neoplasia, tumors, infection, and trauma [1,2,3]. The risk of lumbar spondylolisthesis and spondylosis is higher among the aging population and is considered as a major cause of lower back pain that severely impacts daily life [4]. Lumbar interbody fusion (LIF) is a surgical intervention to overcome degenerated lumber segments and its decompressed neural components as well as related facet joints abnormalities [5]. LIF is mainly performed through removing discs, placing pedicle screws and a bone graft cage in the interbody disc space to promote lumber fusion [6]. Of various LIF techniques, minimally invasive transforaminal lumbar interbody fusion (MIS-TLIF) techniques is preferred, as it minimizes blood loss, spinal neurons damage, and infection [7]. The MIS-TLIF procedure also controls damage to incised tissues and recovery time post-surgery [8,9]. Although MIS-TLIF is favored compared to open TLIF [10], its efficacy and safety depends on surgeon expertise, which could be further enhanced through recent technological developments such as imaging and guiding techniques. The prime objective of fusion is to stabilize degenerated lumber segments through minimizing movement, relaxing spinal nerve, and lowering the pain [11]. The fusion stability is critically affected by suitable positioning of the pedicle screw [12], and therefore, continuous effort is being made to improve screw design. The selection and placement of the interbody cage material also impacts the stability and efficacy of TLIF [13,14,15]. Progresses have been made to improve the surgical procedure, materials, and instrument to reduce the length of stay and radiation exposure during MIS-TLIF, as prolonged exposure may adversely affect patient’s health and recovery time [12]. Owing to minimal skin incision, reduced muscular dissection and retraction of thecal sac, limited nerve root injury, and the advancement in microscopy techniques and tubular retractor systems, MIS-TLIF is performed at a large scale [16]. Although the technical and instrumental developments have significantly improved the learning curve and surgical efficacy [17], the potential of regenerative therapeutic biomaterials for spinal disorders is being explored to achieve the utmost clinical outcomes [18]. The regenerative intervention provides long-term therapeutic relief and also provides opportunity to control the degenerative progression [19]. Additionally, biomaterials can also act as carrier for delivering regenerative cells and osteoinducing factors in interbody and intervertebral disc space [20,21]. Under the umbrella of regenerative therapies, stem cells and platelet-rich plasma (PRP) possess immense potential to improve the therapeutic safety and efficacy of MIS-TLIF [22,23,24].

## 2. MIS–TLIF: The Surgical Procedure

**Figure 1:**
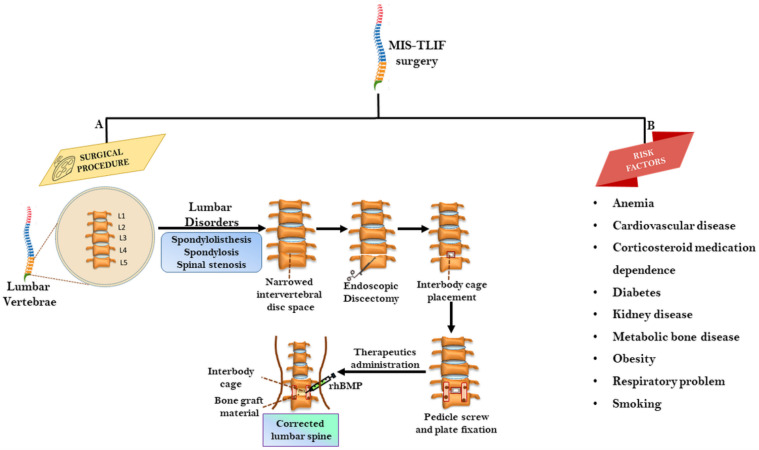
Overview of MIS-TLIF surgery. (**A**) The surgical procedure for correcting lumbar spinal disorders, which includes endoscopic discectomy, interbody cage placement, pedicle screw and plate fixation and administration of therapeutic rhBMP or bone graft material (**B**) Risk factors limiting TLIF surgery involves anemia, cardiovascular disease, corticosteroid medication dependence, diabetes, kidney disease, metabolic bone disease, obesity, respiratory complications an smoking. MIS-TLIF: minimally invasive transforaminal lumbar interbody fusion, rhBMP: Recombinant human bone morphogenetic protein.

The safety and efficacious outcomes of MIS-TLIF much rely on technical expertise and surgeon experience. The surgical procedure initiates with fluoroscopic imaging to identify the level of nerve decompression, degree and positioning of lumber spine, which has been described in previous studies [7,8,25]. Generally, MIS-TLIF is a sequential procedure of incision, discectomy, and decompression/fixation, bone grafting, and fusion (Figure 1A).

The endoscopic approach in MIS-TLIF has been further added to improve minimalism (the minimal invasive nature) by performing a lumbar lateral decompression and interbody fusion via the transforaminal approach, using thin tubular devices under endoscopic visualization. Depending on the endoscopic system applied, the surgical techniques can be distinguished into percutaneous endoscopic (working-channel), biportal, and microendoscopic TLIF [26,27,28,29]. According to a meta-analysis, endoscopic TLIF could significantly improve the preoperative scores for leg and back pain; however, conducting long-term and randomized controlled trials are suggested to reach the conclusion. Meanwhile, for the treatment of lumbar disc herniation and biradicular symptoms, both percutaneous endoscopic lumbar discectomy and MIS-TLIF showed positive clinical outcomes [30]. It has been further noted that compared to MIS-TLIF, percutaneous endoscopy may be advantageous as it be conducted under local anesthesia with a rare chance of adjacent segment disease. However, percutaneous endoscopy is associated with a comparatively low success rate and satisfaction as well as a higher rate of postoperative long-term chronic low back pain and the chance of recurrence. Taken together, expert opinions have concluded that achieving a definitive full-scale decompression and sufficient interbody fusion remains limited in the endoscopic surgical field, which could be overcome through advancements in surgical instruments and optics [31].

### 2.1. Analyzing Factors Influencing Fusion Rate in MIS-TLIF

Following the MIS-TLIF procedure, improvement in the interbody fusion rate minimizes hospitalization time, efficacy, and safety. Fusion is also impacted by bone materials (autograft/allograft or synthetic), cage size [32], osteoinductive molecules, viable cells, and regenerative materials [33]. On the contrary, meta-analysis has also reported that irrespective of the graft material, the fusion rate in MIS-TLIF is very high [34]. Interestingly, no significantly improved fusion rate by rhBMP-2 during PLIF and TLIF has also been reported [35]. The fusion rate could be further improved through employing recombinant human bone morphogenetic protein (rhBMP) along with autologous local bone and bone extender; however, rhBMP is associated with adverse effects such as symptomatic ectopic bone, radiculitis, and vertebral osteolysis, which limits its wide application [34,36]. However, the autografts are relatively preferred owing to their enhanced immunocompetency in suppressed risk of immune rejection and infection [37]. Attempts have also been made to establish correlation between the mixture ratio of autograft and fusion rate in MIS-TLIF. According to an important report, at least 12 mL of bone graft is crucial to MIS-TLIF to reach an adequate bone fusion [38]. Although no statistically significant relationship was found, the fusion rate significantly enhanced to 92.5% in graft volume of more than 12 mL. A few studies indicated that instead of cage materials, their positioning influences the interbody fusion rate and stability of lumbar [39]. Of note, compared to a bullet cage, steerable cage placement may be relatively efficacious in restoring focal lordosis [40]; however, fusing intervertebral segments should be clinically observed to establish their role [41]. Therefore, supplementing bone graft in disc space and its cage interaction might improve lumbar fusion. Studies have also suggested that compared to unilateral, the bilateral posterior screw fixations are preferred in lumbar interbody fusion [42]. In contrast, the perioperative performance of unilateral fixation seems better than bilateral; however, bilateral fixation is more effective in providing long-term relief after MIS-TLIF [43]. Interestingly, no significant difference has also been reported in the fusion rate of unilateral and bilateral fixation [44]. The fusion rate for both the cortical bone trajectory (CBT) screws and normal pedicle screw have been demonstrated to be similar, yet the clinical outcome of the CBT screw is better than the pedicle screw in terms of less pain with high satisfaction [45,46]. The CBT screw also reduce blood loss, hospital admission time, and incision length; however, the lack of studies on cage position and its impact on unilateral or bilateral fixation limits the understanding the relation between lumbar stability, pattern of screw fixation, and its role in the interbody fusion rate [41,47]. Furthermore, smoking behavior also significantly influences the efficacy and safety of MIS-TLIF [39], as the consumption of over 10 cigarettes per day inhibits bone healing and promotes non-union of lumbar [48,49]. Smoking also contributes to pseudoarthrosis and operative complications such as infection and dysphagia [48]. Attempts have been further made to develop an interbody cage with piezoelectric composite materials to improve the fusion rate [50,51]; however, in the aged patients, although direct current simulation is ineffective in improving lumbar fusion, the enhanced functional outcomes have been demonstrated [52,53]. This is also supported by a systematic review and meta-analysis that revealed no impact of bisphosphonate drugs on the lumbar fusion rate, but it could suppress the risk of vertebral fractures [54]. In a rat spinal arthrodesis model, the intermittent intervention of human parathyroid hormone (1-34) accelerated bone graft resorption and fusion rate on day 14, and it eventually yielded a structurally superior lumbar fusion mass [55]. In addition to these therapeutic approaches, the surgical expertise of a surgeon could also reduce operation time and radiation exposure, leading to improved pain and neurological symptoms of back and limbs of an MIS-TLIF patient [56]. Taken together, the above-mentioned factors indicate their contribution in improving MIS-TLIF outcomes, which have been comprehensively reviewed in the next section.

### 2.2. Radiologic and Clinical Outcomes of MIS-TLIF 

The extensively assessed clinical outcomes of patients who underwent MIS-TLIF offer a clue to further improve condition. Based on the Oswestry disability index (ODI) of a propensity matched cohort study, compared to open TLIF, the MIS-TLIF could effectively ameliorate pain post 2 years treatment, [57], indicating the prolonged therapeutic efficacy of MIS-TLIF. Similarly, MIS-TLIF may also effectively improve life quality with an enhanced fusion rate, a reduced rate of secondary surgical intervention, and fewer complications [58]. In a prospective, multi-institutional study on lumbar spine surgery in morbidly obese patients, improvement in back and leg pain, physical component score/mental component score, and life quality was observed with no divergence in post-operative complications between MIS-TLIF and open TLIF [59]. While in the other study, compared to open TLIF, MIS-TLIF improved blood flow in the incised skin with no change in levels of blood oxygen and hemoglobin [60]. In a long-term clinical follow-up for treatment of one-segment lumbar disc herniation, MIS-TLIF predominated conventional TLIF in efficaciously reduced blood loss, intramuscular pressure, operative time, mean return to work time, as well as visual analogue score (VAS) and ODI indices, implying pain relief [61]. Nonetheless, both surgical interventions were found comparable with respect to fusion rate, lordosis, and sacral slope. In a notable report, the MIS-TLIF and open posterior lumbar interbody fusion (O-PLIF) revealed a comparable fusion rate and clinical outcomes with nearly equal complication rate [62]; however; the blood loss, recovery time, and frequency of adjacent segment degeneration was lower in MIS-TLIF patients. In line with the above reports, a meta-analysis of 10 randomized and non-randomized clinical trials also confirmed MIS-TLIF-mediated efficacy in lowering hospitalization time period, blood loss, and complications rates as compared to open TLIF (O-TLIF), with no significant difference in operative time, VAS of back pain, and leg pain and ODI [63]. Interestingly, the report also revealed that compared to MIS-TLIF, the O-TLIF may better improvise inter-segmental parameters including anterior disc height and segmental lordosis, but only MIS-TLIF could reduce visible blood loss and hidden risk of hemorrhage group [64]. In another seminal study, although MIS-TLIF was found to be more effective in reducing blood loss, incision size, and lower back pain, the Wiltse-TLIF (W-TLIF) diminished the X-ray exposure and hospitalization time, and no significant difference was found in VAS of leg pain and fusion rate [65]. Similarly, a meta-analysis indicated that MIS-TLIF is also effective in improving ODI, VAS, and reducing the related complications compared to MIS-lateral lumbar interbody fusion (MIS-LLIF), with no considerable difference in fusion rate [66]. 

In recent years, the novel percutaneous endoscopic TLIF (PE-TLIF) technique has been evidenced in reducing surgical trauma, blood loss, and low back pain along with improvement in recovery time when compared to MIS-TLIF [67]. However, PE-TLIF requires stringent follow-up of outcomes indicators and in-depth learning curve of surgical procedure. The return to work after MIS-TLIF very much depends on the preoperative level of ODI, North American Spine Society score for neurogenic symptoms (NASS), numerical pain rating scale, back and leg pain score. The low and high levels of these indices indicate early and delayed return to work, respectively [68]. Interestingly, similar clinical outcomes (improvements in pain, function, and life quality), perioperative complications, fusion rates, and safety of MIS-TLIF have also been reported between elderly and young patients [69]. Based on the above evidence, it could be inferred that MIS-TLIF is an effective surgical intervention over other possible TLIF in providing long-term relief. However, to establish a clear consensus on the supremacy of MIS-TLIF compared to other interbody fusion techniques, more pragmatic randomized clinical trials should be conducted with low risks of systematic and random errors.

### 2.3. Limitation of MIS-TLIF

The primary objective of MIS-TLIF surgery is to minimize incision size and damage risk to nearby soft tissues and blood loss. Although MIS-TLIF is gaining an increasing acceptance due to its efficacy and safety, the success of MIS-TLIF is limited by factors such as patient selection, level of lumbar deformity, prior surgical preparation, and learning curve. The patients need to be screened for potential spinal instability, sagittal imbalance, osteoporosis, and advanced osteopenia, as the existence of these abnormalities is unsuitable for performing lateral fusion [70]. A prior presence of fusion mass, cervical/thoracic deformity, and austere sagittal imbalance also limits the application of MIS-TLIF. The complications such as numbness, paresthesia and weakness, abdominal wall paresis and bowel perforation, lateral incisional hernia, colonic pseudo-obstruction and bowel perforation, and inability to suppress the proximal junctional kyphosis (PJK) and proximal junctional failure (PJF) frequency restrict the wide application of MIS-TLIF. The risks of dural tears aid to additive treatment to address this complication in MIS-TLIF treatment [71]. The difficulties associated with the learning curve such as durotomy, implant misplacement, neural damage, and incomplete fusion complications are variable and need to be addressed through sufficiently improved training of MIS-TLIF [72]. High radiation exposure hazards linked with MIS-TLIF have been reported; however, more extensive studies are required to completely assess the mentioned risk [73]. 

### 2.4. Challenges and Risks in MIS-TLIF

Performing a successful MIS-TLIF is a daunting task among obese and aging patients with existing complications and disorders, as the peri- and post-operative risk is much higher compared to patients with normal body mass index (Figure 1B). The increased adverse incidences such as delayed wound healing, blood loss, urinary and pulmonary complications, prolonged operative time, increased surgical/operative cost, and longer hospital stay have commonly been associated with obese patients [74,75,76,77]. Apart from this, the blood loss anemia [78,79], diabetes, and its complications have been reported to complicate the outcome and length of stay in MIS-TLIF [80]. In contrast, no association between diabetic complications and postoperative complication rates, inpatient length of stay, or direct hospital costs following MIS-TLIF has also been evidenced [81]. In addition, smoking may adversely influence the internal region of the IVD by imparting vasoconstriction, leading to a reduced supply of nutrients and growth factor to disc tissue, which further suppress glycosaminoglycans and cell density [82,83]. While addressing this pathologic condition, MIS-TLIF is effective in reducing blood loss, frequency of short-term pain, complications, and recovery time in comparison to posterior lumbar interbody fusion (PLIF) among obese patients; however, the operative time in MIS-TLIF was much longer than PLIF [84]. In addition, gain in short-form 12 mental and physical composite score gain remained unaffected by the obesity status of MIS-TLIF-treated patients [85]. Prolonged operative time among diabetic and obese patients may increase risk of surgical complications up to 2 years after MIS-TLIF [80,86]. In addition, pseudoarthrosis/nonunion has also been associated with postoperative MIS-TLIF surgery [87]. The surgical outcomes of MIS-TLIF is highly impacted by aging; however, a recent retrospective study in the elderly (>65 years) and younger (<65 years) patients revealed significant improvements in both groups with no significant difference in blood loss, hospitalization stay time period, mobilization, and ODI, VAS, and Wang’s score. This indicate that the aging is not a deterrent to outcome and should not be considered as a contraindication to perform MIS-TLIF in the older population [88]. In another retrospective study, the efficacy of grade 1 fusion and complication rates were identical in middle- and old-aged patients at the end of 2-year follow-up [89]. However, older patients experienced much longer time in body movement and hospitalization with comparatively lesser improvement in ODI and SF-36 physical function scores, although the NASS, VAS, and SF-36 mental scores were equivalent among both age groups. A prospective study also mentioned no observed significant difference in pain (back and leg), ODI, SF-36 score, as well as fusion rate among aged and young patient groups two years post-surgery [69]. When clinical and surgical differences were minimized, the MIS-TLIF seemed equally effective for aged patients without any considerable post-operative complexity. While performing MIS-TLIF, the violation of the superior facet-joint is a major risk factor. It has been further observed that body mass index (BMI) ≥ 30 kg/m^2^ and the placement of a pedicle screw at L5 may be independent risk factors for superior facet-joint violation during MIS-TLIF and could occur mainly in hypertrophic facet joint or coronal orientation [90]. These previous reports suggest that pre- and post-operative challenges and risks should be carefully assessed to determine the safety and efficacy of MIS-TLIF. 

## 3. Currently Employed Surgical Materials for Bone Grafting and Fusion 

**Figure 2:**
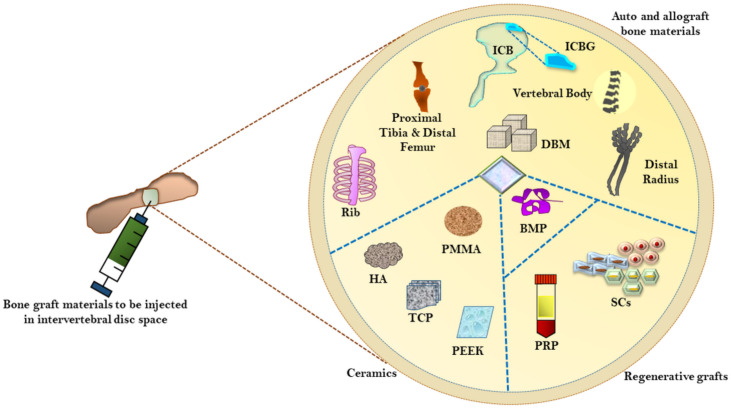
Overview of bone graft materials available for interbody fusion. Currently, the three categories of bone graft materials are being explored, which include ceramics, auto/allografts, and regenerative grafts. BMP: Bone morphogenetic protein, DBM: Demineralized bone matrix, HA: Hydroxyapatite, ICBG: Iliac crest bone graft, PRP: Platelet-rich Plasma, PEEK: Polyetheretherketone, PMMA: Polymethylmethacrylate, SC: Stem cells, TCP: Tricalcium phosphate.

The source of bone grafting is considered essential for successful interbody spinal fusion. Current bone graft alternatives include autografts, allografts, demineralized bone matrices (DBM), and ceramic materials (Figure 2) [37], which possess osteoconductive, osteoinductive, and osteogenic functions and promote bone fusion [91]. The use of autograft reduces the risk of immune reaction, materials rejection, and disease transmission. The iliac crest-derived autografts is considered a common source; however, their harvest results in pain, prolonged operative time, and insufficient amount [91]; whereas allograft seems a viable alternative to harvest graft materials in adequate quantity. However, the resultant adverse reactions associated with grafting and acceptance limits its broader application. The other bone graft materials such as ceramics, collagen-based matrices, bone marrow aspirate (BMA), and DBM are also considerable grafting options. Additionally, the bone morphogenetic proteins (BMPs), synthetic peptide, and autologous growth factors could assist in inducing rapid healing. 

### 3.1. Autografts versus Allografts as Bone Material Substitutes 

Porosity-based bone autografts are classified into cortical and cancellous, in which cancellous is comparatively porous [92] and promotes higher revascularization and osteogenesis even though the initial mechanical strength of graft is low [32,93,94]. In contrast, dense cortical bone could initially improve the mechanical strength and stability, even though the revascularization becomes much difficult during bone synthesis. Autografts derived from iliac crest are rich in live cells, osteoinductive growth factors, and channels necessary for vascularization, which could promote effective lumbar fusion [95]. A retrospective study with 75 consecutive cases showed that iliac crest bone graft (ICBG) could effectively aid in achieving a higher rate of spinal fusion without any major perioperative or latent complications and transfusion [33]. Furthermore, no significant difference in postoperative pain/disability and blood loss was found between BMP-2 and ICBG-treated patients. Although the therapeutic significance of ICBG is widely recognized, the complications pertaining to ICBG harvest, such as infections, fractures, seromas, graft site hernia, nerve injury, sensory disturbances, increase in operative time, hematomas, and donor site morbidity, limits application of ICBG as bone grating materials [37]. In addition to iliac crest, the proximal tibia, rib, fibula, distal femur, distal radius, and vertebral body are also identified sources of autografts [37,96]; however, these are associated with increased blood loss, operative time, and donor site morbidity. Further, local bone graft extracted from laminae seems to be a viable alternative for ICBG for providing effective lumbar infusion [97,98]. During posterior lumbar interbody fusion (PLIF), the local morselized bone graft effectually reduced operation time, blood loss, and hospitalization time [99]; whereas in TLIF, it resulted in definitive union with only one case of possible pseudoarthrosis [100]. Therefore, local body bone graft has not been suggested for high grades slippage with anterior vertebral translation over 25%, and it was also found to be ineffective in imparting bone fusion [101].

Apart from autografts, the allografts have been studied for their role in interbody fusion. Allografts are harvested from the cadaverous tissue of the donor after surgical intervention such as hip replacement [32], and they could be implanted during MIS-TLIF surgery, the efficacy of which may be enhanced via expandable meshed containment device [102]. While lumbar interbody fusion surgery for segmental sagittal lordosis, a synergistic application of allograft and recombinant human BMP-2 (Rh-BMP2) have improved pain, ODI score, and reduced blood loss [103]. The allografts are also osteoinductive and osteoconductive and provide necessary scaffold support for bone formation [104]. However, the lack of sufficient viable cells may significantly reduce its osteogenic potential [105]. The allogenic bone grafts are mainly synthesized in the form of demineralized bone matrix, cortical grafts, bone segments, morselized/cancellous chips, and cortical grafts [106]. The low-cost availability of allograft and reduction in donor site morbidity encourages its applicability as an alternative for autograft, but it may result in complications such as risk of disease transmissions and viral infection [106]. Notwithstanding, the cautious inspection of allograft materials may reduce the likelihood of these adverse reactions. 

### 3.2. Demineralized Bone Matrix (DBM)

**Figure 3:**
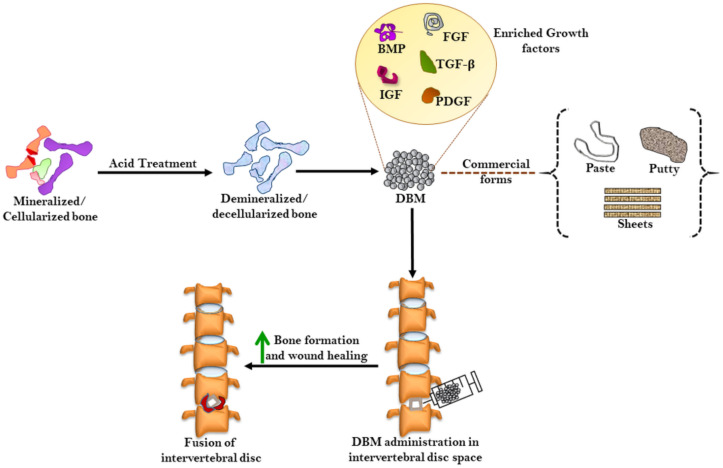
Strategies for synthesizing DBM. Following mild acid treatment, DBM is produced that contains various growth factors for tissue repair. The commercial forms of DBM include paste, putty, and sheets, which could be administered in an intervertebral disc space to assist wound healing and spinal fusion. DBM: Demineralized bone matrix, IGF: Insulin-like growth factor, PDGF: Platelet-derived growth factor, TGF: Transforming growth factor, FGF: Fibroblast growth factor.

DBM is extracted through mild acid treatment of human bone resulting in a protein-rich matrix mainly containing collagen along with growth factors, such as bone morphogenetic protein (BMP), fibroblast growth factor (FGF), insulin-like growth factor (IGF), platelet-derived growth factor (PDGF), transforming growth factor-β (TGF-β), some calcium-related molecules, and traces of inorganic phosphates and cellular debris (Figure 3) [32,107,108]. DBM constitutes around 14.4% as graft materials in arthroplasty, 82% in trauma surgery, and 89% in foot/hand surgery [109]. The demineralization process particularly decalcification from DBM destroys its antigenic characteristics, thereby minimizing the host graft inflammatory response [110]. Depending on the microenvironment into which the DBM is planted, the mechanism of osteo- or chondrogenesis differs. This might be attributed to the specific bone or cartilage formation pathways resulting from mesenchymal stem cells in subcutaneous tissues and calvaria having a high population of stem cells with different receptors, which may selectively interact to osteo- or chondrogenic proteins [107]. DBM is commercially available in the forms of putty, paste and sheets; however, their variation in growth factors and total proteins has been observed among different products and brands such as DBX, Grafton and Allomatrix [111]. Collagen as a major component of DBM exerts osteoinduction, whereas the BMP provides functional support to the matrix, while its degradation releases growth factors at the wounded site and accelerates bone synthesis. The varying BMP concentration in commercial DBM greatly impacts its clinical efficacy [37]. In an athymic rat model, a large variation in fusion performance of DBM and demineralized bone fiber (DBF) of seven commercial products (StrandTM Family, Propel^®^ DBM Fibers, Vesuvius^®^ Demineralized Fibers, Optium^®^ DBM Putty, Grafton^®^ DBF, Grafton Flex, and DBX^®^ Putty) was observed, and outcomes indicated a detrimental influence of the carrier on the fusion performance; hence, DBM allografts should be carefully selected [112]. Research has also shown that different commercial DBM products (Grafton Putty, DBX Putty, and AlloMatrix Injectable Putty) may results in varying magnitude of new bone formation and radiographically evident spinal fusion [113]. DBM putty combined with a single local application of zoledronic acid could also enhance new bone synthesis during posterolateral fusion [114]. On contrary, the inhibitory effect of DBM on the solid bone fusion of spine has also been evidenced [115]. Taken together, although various pre-clinical and clinical studies have confirmed the graft extender/enhancer role of DBM in spinal fusion, their varying efficacy compels the need for extensive studies to clearly establish its clinical potential [116]. 

### 3.3. Ceramics as Bone Grafting Materials 

Ceramics are mainly synthesized via a temperature-controlled process known as sintering, which offers an opportunity to modify the structure, porosity, and bio-degradability [32,117]. Ceramics could also minimize the risk of infection and disease transmission as well as increase shelf-life and the availability of implantable materials [105,106]. However, compared to ICBG, less efficient bone extender in the terms of brittleness, instability for cortical implants, low compression and shear strength limits the ceramic application in bone fusion [118]. Calcium-based materials such as calcium phosphate, tri-calcium phosphate, calcium sulfate, hydroxyapatite (HA), silica-substituted calcium phosphate, and bioglass are mainly used as bone graft with osteoconductive characteristics [32,118]. Although ceramic porosity could promote the adhesion, proliferation, and differentiation of mesenchymal stem cells into osteoblasts; however, a high degree of ceramics porosity restricts the mechanical strength and resorption rate [119,120,121]. Recently, the applications of ceramics and related scaffolds have increased in spinal surgeries, and in particular, they have been employed as a bone graft extender for lumbar spine arthrodesis leading to an 86.4% improved spinal fusion rate [121]. Furthermore, HA present in human bone is most common ceramics, which could serve as an excellent carrier of osteogenic cells and growth factors and promote bone fusion [122]. Thus, in the presence of an appropriate growth factor, HA can also play both an osteoconductive as well as an osteoinductive role during bone fusion. The augmentation of a pedicle screw with HA may improve the screw construct in the upper level of fixation in MIS-TLIF surgery [123]. Similarly, in a porcine model, the use of a kidney-shaped nano-hydroxyapatite/polyamide 66 cage provided an efficacious internal fixation and spinal stability after TLIF surgery [124]. HA could also be coated with polyetheretherketone (PEEK) to improve cell adhesion, proliferation, differentiation, and the bone–implant contact ratio [125]. Moreover, the mixing of autograft with bone marrow aspirate and nanohydroxyapatite could result in lumbar bone fusion equivalent to iliac crest graft. However, compared to granular HA, block HA may be more effective in bone implant due to the better porosity requirements for bone growth [126]. Hence, the physical structure of HA also impacts its efficacy in bone fusion, and the clinical outcomes of the HA-DBM composite has been found to be similar to autografts in lumbar interbody fusion, even in the aging and low bone mineral density patients [127,128]. Another ceramic material—i.e., tricalcium phosphate (TCP), found in mainly α and β crystalline forms—has been reported with a higher solubility, biodegradability, and bone regeneration rate than HA; however, with low mechanical strength [129,130]. In addition to being osteoconductive, TCP is also osteoinductive with better cell resorption property. The osteoinduction and porosity of TCP could be modified through coating or mixing impurities such as HA and β-calcium pyrophosphate [131]. During in vitro study, TCP exerted no adverse effect on the growth and morphology of rat osteoblasts [132], and in a clinical trial, the fusion efficiency, lower pain, and side effects were found to be comparable to autograft [133]. The filling of a fusion cage with TCP cement may improve the clinical outcomes without any surgical complication in lumbar interbody fusion [134]. In a clinical retrospective cohort study, a more effective fusion by TCP has been found to be higher when compared to a titanium cage packaged with HA within 1 year of surgery [135]. However, no significant difference in fusion was observed after 2 years. Similarly, ultraporous TCP has been clinically observed as an effective bone void filler in spinal arthrodesis and can reduce morbidity [136]. The combined bone graft materials of β-TCP and HA with bone marrow aspirate have been found safe and efficacious in lateral interbody spinal fusion with significantly reduced blood loss [137]. It is important to note that an increase in the porosity of TCP improves wicking and hydrophilic properties resulting in an enhanced availability of nutrients, growth factors, and osteogenic cells. The ability of ultraporous TCP in inducing interbody fusion has been already demonstrated in anterior lumbar interbody fusion (ALIF) or posterior lumbar interbody fusion (PLIF) [138]. Similarly, calcium sulfate (CS), Si-calcium phosphate (Si-CaP), polyetheretherketone (PEEK), polymethylmethacrylate (PMMA), and bioglass are also considered as alternative ceramics for interbody fusion [37,118]. Interestingly, the CS has not been found to be effective in promoting lumbar fusion in rabbits [139]; however, its impregnation with antibiotics may promote bone cells attachment to graft to achieve the desired biocompatibility, osteoconductive, and osteoinductive properties [140]. The micro-vessel density in tibial metaphysis could also be enriched with CS [141], and when supplemented with autograft or bioglass may prove to be an effective bone graft extender [142] with increased strength and degradability during spinal fusion [143]. A composite of multi-amino acid copolymer/nanohydroxyapatite/calcium sulfate can also be employed to attain required stability, bone fusion, and integration [144]. Apart from this, polymethylmethacrylate (PMMA) is an economical, inert, and biocompatible material which is mainly used as cementing agent in joint replacement, and it has also been considered as a potent candidate in lumber fusion [145]. The PMMA when augmented with fenestrated pedicle screw postoperatively improved VAS and the ODI score in the MIS-TLIF of aged patients without deterioration in cementing material and pedicle screw with the absence of any significant adverse event [146]. In a recent study on MIS-TLIF in a goat interbody lumbar fusion model, the mineralized collagen–PMMA bone cement with a unilateral pedicle screw achieved the desired osteogenesis and bone growth [147]. Polyetheretherketone (PEEK) is another ceramic employed in spinal fusion, but the fusion efficiency has not been found to be significantly different from titanium cage [148]. Subsequently, composites of HA/PEEK and HA/PEEK with nano-TiO_2_ have improvised the PEEK potential for its utilization in the interbody fusion [149,150] and revealed with more bone synthesis at the fusion site as well as ingrowth into porous end plates in an ovine model [151]. These preclinical and clinical bodies of evidence strongly support the combinatorial potential of TLIF and ceramics in the near future.

### 3.4. 3D Printing in Spinal Surgery

Recent developments in the additive manufacturing technique also known as three-dimensional (3D) technology have opened new horizon for printing spine-associated biomedical products such as vertebral implants [152]. Three-dimensional (3D) printing also facilitates introducing a complex internal geometry of interbody cages with the desired roughness and porosity to improve bone remodeling and increase the efficacy and safety of surgical intervention [153,154]. Moreover, the 3D printing not only provides flexibility to mold a complex structure but also control over pore size, leading to improvement in osteointegrity, osteoconductive, and osteoblastic activity [154,155,156]. Compared to computed tomography (CT), a 3D-printed patient-specific navigational template is a cheaper and more precise methodology to direct the screw placement and is not influenced by the patients’ intraoperative positional changes. In 3D printing technology, melted materials are placed layer-by-layer, using three methods including fused deposition modeling, selective laser sintering, or stereolithography [157]. Available choices of 3D printing materials include titanium, poly-ε-caprolactone, PEKK, and tantalum [158,159]. A single surgeon-based prospective study using 3D-printed lamellar titanium cage packed with silicate-substituted calcium phosphate (SiCaP) in TLIF and lateral lumbar interbody fusion (LLIF) showed an excellent solid fusion rate and cage integration at the vertebral body interface without any event of screw loosening [154]. The clinical outcomes such as VAS score and ODI were also found to be improved. However, SiCaP might cause an illusion of bone fusion, which could be critically analyzed through biopsy. Three-dimensional (3D) printing technology can be a critical tool to design a preoperative model and a training/education tool for clinicians as well as patients [156]. The 3D-printed spine could also facilitate freehand pedicle screw placement and improve the surgical outcomes while correcting spinal deformity without any vascular or neurological complications [160]. The possibility of enhanced rates of accurate screw positioning and higher numbers of inserted screws using 3D printing has been suggested due to increased safety, specifically at apical levels [161]. The synergistic application of pedicle guider technology to 3D printing has been shown to enhance surgical efficacy and reduce the operative time during the treatment of congenital scoliosis [162]. This suggests that the TLIF surgery-mediated outcomes may be upgraded by 3D and pedicle guider technology. Compared to the standard free-hand technique, the MySpine^TM^ comprising a low dose of CT scan and 3D-printed patient-specific guide system may significantly reduce the incidence of pedicle screw malpositioning, intraoperative radiation exposure, and surgical time of the implantation phase [163]. Taken together, 3D printing seems a potential alternative to improve the surgical outcomes by reducing the operating time through accurate pedicle screw positioning, stimulating osteointegrity, osteoconductive, and osteoblastic activities. Therefore, subjective approval of 3D printing-based surgery needs to be considered for establishing the role of 3D printing in spinal surgery. 

### 3.5. Bone Morphogenetic Protein-2 (BMP-2) 

BMPs are growth factors that belong to the transforming growth factor-β (TGF-β) superfamily and promote cell proliferation, differentiation and the maturation of mesenchymal stem cells (MSCs) into chondrocytes and osteoblast-like cells [105,164]. As a result, BMPs seem to acquire osteoinductive characteristics, and therefore, they have been studied for their bone healing and growth potential [32]. To circumvent post-operative morbidity and complications after harvesting ICBG, the recombinant human BMP-2 (rhBMP-2) is employed as a potent bone graft enhancer [165]. Clinically, rhBMP-2 has been proven not only to improve bone fusion but also improve recovery in back pain [166]. However, a critical review on the emerging safety concerns of clinical trials of rhBMP-2 in anterior interbody lumbar fusion indicated higher rates of implant displacement, subsidence, infection, urogenital events, and retrograde ejaculation after employing rhBMP-2, whereas posterior lumbar interbody fusion was accompanied with poorer global outcomes including radiculitis, ectopic bone formation, and osteolysis [167]. In a meta-analysis of randomized clinical trials, although the efficacy of rhBMP-2 was found to be superior to ICBG in terms of significantly decreasing the risk of fusion failure at all-time intervals, the recommendations from the Food and Drug Administration and further independent reports still suggest the security risks in using rhBMP-2 [168]. Consequently, prospective clinical studies even attempted to synergistically employ a low dose of BMP-2 and autograft, which resulted into high rate of early fusion but still encountered BMP-associated adverse events such as asymptomatic heterotopic ossification and perineural cyst formation [169,170]. On the contrary to this, a retrospective cohort study reported a high fusion rate by using low-dose rhBMP-2 in the MIS-TLIF procedure with a very rare complication rate (0.6%) [171]. It has also been advocated that compared to autograft, the off-level rhBMP-2 might be more effective in bone fusion after TLIF; however, radiculitis and seroma were prevalent among the rhBMP-2 group with the complication rate similar to autografts receiving patients [172]. In a seminal study, rh-BMP-2 did not retard the rate or number of reoperations of pseudarthrosis after MIS-TLIF [173]. The BMP dose/level also significantly predicted the magnitude of fusion and the 1.0 mg/level has been suggested as a minimally effective dose [174]. Meanwhile, a seminal meta-analysis reported the lowest minimally effective dose for fusion for PLIF and TLIF as 1.28 mg/level with no significant difference of fusion and complication rates with varying doses of BMP [175]. Based on the collective evidence, we infer that BMP-2 could be an effective bone graft alternative, even though conflicting results related to complications need to be addressed. 

## 4. Regenerative Therapeutic Approaches for Bone Grafting and Fusions in MIS-TLIF

### 4.1. Stem Cells and Cellular Bone Matrices in Bone Grafting

Stem cells are undifferentiated and have potential to differentiate into any of endoderm, ectoderm, and mesoderm cell lineages; however, it depends on the specific microenvironment and cell source or type. In recent decades, the differentiation potential of stem cells has extensively been explored to develop regenerative therapies for various disorders. It has been reported that as a bone graft, the bone marrow aspirate (BMA) rich in mesenchymal stem cells (MSCs) and osteogenic factor could efficaciously stimulate spinal bone fusion [176,177]. The osteogenic potential of bone marrow stem cells (BMSCs) also plays a key supportive role in the therapeutic effect of BMA in bone fusion. The in vitro data reflect that BMSCs could retard the matrix degeneration rate in nucleus pulposus (NP) through downregulating and upregulating the nuclear factor kappa B (NF-κB) and TGF-β pathway, respectively [178]. Furthermore, an allogeneic bone graft rich in MSCs popularly known as cellular bone matrices (CBMs) have also been synthesized and are commercially available; however, their efficacy and safety remains to be validated by the non-industry sponsored studies [179]. On the contrary, no significant contribution in bone fusion of the cellular component of two commercial CBMs has also been reported [180]. Therefore, the extensive clinical studies are highly needed to reach a clear consensus.

In the recent years, the allogeneic undifferentiated rabbit BMSCs [181] and bio-conditioned gelatin sponge with osteogenically enhanced human BMSCs [182] have been shown to enhance bone fusion without any major immunological adverse challenge in rabbit and rat models, respectively. Alongside these novel attempts, hyperbaric oxygen therapy exerts no significant effect on MSCs for spine fusion; however, it has been suggested to optimize oxygen tension and pressure to maintain and enhance the osteogenic ability of preconditioned MSCs [183]. Furthermore, bone fusion and regeneration could be improved through implanting a composite of autologous BMSCs and calcium phosphate ceramic [184], and when enriched with β-TCP, it may exert an effective spinal fusion without any major adverse event [185]. In a long-term clinical retrospective study, BMSCs concentrate harvested through selective cell retention technology augmented spinal fusion by improving the VAS, ODI score, and mean disability index [186].

In addition to BMSCs, the adipose-derived stem cells (ADSCs) are another viable regenerative alternative for spinal fusion. In a rabbit model of traumatic intervertebral disc (IVD) established by percutaneous needle puncture, the administered ADSCs enhanced the secretion of the extracellular matrix and ossified damaged cartilage in nucleus pulposus [187]. In a novel attempt, autologous ADSCs differentiated with DBM in a scaffold-free osteogenic three-dimensional (3D) graft improved VAS and ODI scores along with confirmed grade 3 fusion without adverse events [188]. Owing to their osteoinductive potential, ADSCs as cellular vehicles for rhBMP-2 delivery may enhance posterolateral spinal fusion by the formation of a large fusion mass without decortification [189]. To induce new bone synthesis and spinal fusion, a minimum period of 7 days expression of rhBMP-2 in MSCs has been suggested [190]. In a comparative study, both the BMP-2-producing ADSCs as well as BMSCs established by adenoviral gene transfer induced sufficient posterolateral spinal fusion in rats [191]. It is also notable that the immune system does not negatively impact the capacity of allogeneic ADSCs to mediate tissue generation in vivo; hence, it could be safely employed to replace or restore damaged tissues and promote spinal fusion [192,193]. Studies have also shown that the radiolucent poly(l-lactide-co-caprolactone) scaffold popularly known as PLCL could facilitate vasculogenesis and rapidly embed ADSCs, to facilitate its proliferation and differentiation into osteogenic phenotype [194,195]. These studies have evidenced that compared to bioceramics, the radiolucent nature of PLCL scaffolds may be advantageous in monitoring in vivo bone formation during spinal fusion via plain radiography. This was further supported by an in vitro study showing that the seeded human ADSCs on a highly porous titanium trabecular spinal cage may promote the differentiation of osteoblast-like cells, leading to a cage structure similar to cancellous bone [196]. The ADSCs could also be synergized with dimethyloxaloylglycine to stabilize levels of hypoxia inducible factor-1α (HIF-1α), resulting in improved angiogenesis, osteogenesis, and spinal bone fusion [197]. Additionally, the composite materials consisting of collagen matrix, nano-HA, collagen, and polylactic acid in the presence of ADSCs have been proven effective in inducing posterolateral lumbar spinal fusion in rabbits [198]. The composite material such as strontium substituted beta-tricalcium phosphate (β-TCP) has been found more inductive toward BMSCs than ADSCs in a rat model [199]. Mechanistically, this may be mediated through overexpressed WNT1-inducible-signaling pathway protein 1 (WISP-1) in human perivascular stem cells promoting osteogenesis rather than adipogenesis [200]. Thus, the regulation of WISP-1 could be assistive in promoting bone fusion in spinal columns. Apart from BMSCs and ADSCs, the human neural stem cells or neural progenitor cells (NPCs) have also been demonstrated to be multipotent with self-renewal and differentiation potential into central nervous system cell types. This has been evidenced by the intramedullary transplantation of human central nervous system stem cells into perilesional tissues above and below the thoracic and cervical region in spinal cord injury patients and found to be safe and feasible at a defined dose [201]. Stem cells derived from IVD components such as annulus fibrosus, nucleus pulposus, and cartilage endplate have been further reported as potential regenerative cell sources for spinal fusion [202]; however, extensive studies are required to characterize and assess their pre-clinical and clinical promises. Interestingly, when compared to BMSCs or ADSCs, human umbilical cord tissue-derived mesenchymal stem cells (UCMSCs) may serve as a viable source for allogeneic applications due to their low immunogenicity and ability to exhibit localized immunosuppression [203]. Notably, a remarkable analgesic behavior of UCMSCs have been clinically revealed through significantly reduced VAS and ODI scores while treating discogenic low back pain [204]. However, while selecting a source of stem cells like olfactory mucosal cell autograft, one should be highly cautious, as its transplantation might lead to intramedullary lesion, edema, and accumulation of mucinous mass within the spinal cord [205].

### 4.2. Exosome-Mediated Bone Regeneration and Spinal Fusion

Alongside stem cells, their derived exosomes, which are rich in cell-communication molecules, could mediate repair and regenerative effects via paracrine signaling [206,207]. These entities are extracellular vesicles with size ranging from ≈40 to 160 nm (average ≈100 nm) in diameter, and they contain nucleic acids, lipids, proteins, amino acids, and metabolites [208]. Exosomes could exhibit comparable therapeutic consequences and functional characteristics as stem cell administration without any adverse events [209,210]. In a recent study, BMSC-derived exosomes have been demonstrated to inhibit microglial activation, A1 neurotoxic reactive astrocytes, neuroinflammation, neuronal cell death, glial scar formation, and lesion volume, and they promoted angiogenesis, axonal regeneration, and functional behavioral recovery in traumatic spinal cord injury in rats [211].The nucleus pulposus cells (NPCs)-derived exosomes also possess the potential to promote BMSCs proliferation and its differentiation into NPCs-like cells [212]. MSCs exosomes could ameliorate advanced glycation end products (AGEs)-induced ER stress in human NP cells through AKT and ERK signaling, leading to inhibited IVD degeneration [213]. Further, a pre-clinical study in mice found that miR-410-derived from MSCs exosomes played critical anti-pyroptosis and promotes recovery from IVD degeneration [214]. Thus, these clinical evidence demonstrate the potential of stem cells’ exosomes in promoting bone fusion after TLIF surgery.

### 4.3. Platelet-Rich Plasma (PRP)-Mediated Bone Grafting and Regeneration

**Figure 4:**
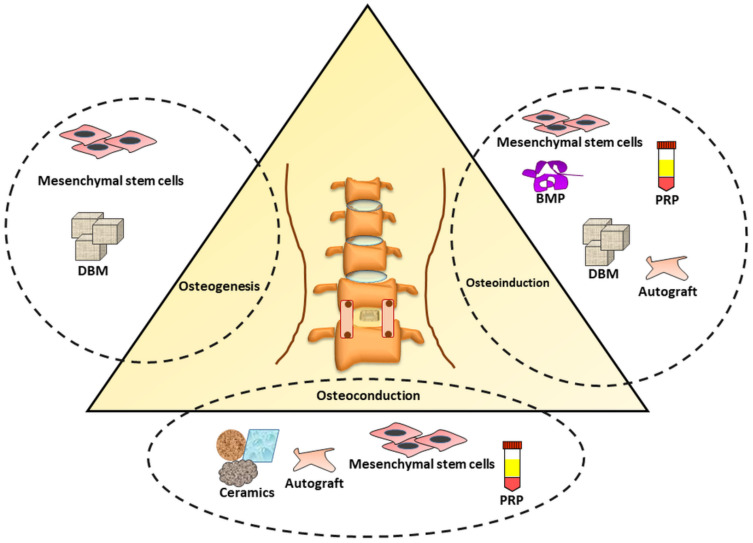
Osteogenic (bone synthesis), osteoinductive (recruiting and stimulating immature cells into preosteoblasts) and osteoconductive (bony growth on a surface) characteristics of bone grafts materials in TLIF-mediated reconstruction. BMP: Bone morphogenetic protein, DBM: Demineralized bone matrix, PRP: Platelet-rich Plasma.

PRP is a concentrate of platelets and other growth factors, which have been implicated as a potent biomaterial in regenerative medicine. In the PRP, the presence of platelet-derived biomaterials (PDB) such as epidermal growth factor (EGF), vascular endothelial growth factor (VEGF), epithelial cell growth factor (EGR), hepatocyte growth factor (HGF), insulin-like growth factor (IGF), platelet-derived growth factor (PDGF), platelet-derived angiogenesis factor (PDAF), platelet factor-4 (PF-4), transforming growth factor (TGF), Il-1, IL-6, and TNF-α have been reported to initiate and participate in the progression of bone fusion and healing [215,216]. Recent pre-clinical and clinical studies have also implicated the potential of PRP in promoting bone fusion during lumbar infusion surgeries. In the SD rats, the synergistic impacts of PRP and HA have been demonstrated to significantly improve interbody spinal fusion by suppressing inflammatory neuropeptide in sensory neurons innervating the discs [217]. In a novel attempt, the frozen-PRP enabled earlier bone union with enhanced bone synthesis and trabecular bone remodeling with more trabecular branches and biomechanical rigidity/strength comparable to freshly employed PRP with BMP in a posterolateral spinal fusion surgery rat model [218]. In the posterolateral lumbar arthrodesis rat model, the PRP led to an increased bone formation and decreased bone fusion time; however, no beneficial impact on suppressing inflammatory pain was evidenced [219]. Based on these positive preclinical outcomes, the conducted clinical reports have also concluded that PRP could enhance the bone fusion in TLIF surgery with no significant difference between fusion time, pain levels in lower back and leg compared to their control counterpart [220]. In a novel clinical attempt, the heterologous cancellous bone substitute soaked with PRP induced both osteconductive and osteoinductive properties (Figure 4) in composite and enhanced bone fusion rate and density in posterolateral arthrodesis patients [221]. This might be ascribed to the mitogenic activities of PRP on osteoblasts and also in inducing the differentiation of mesenchymal stem cells/progenitors into osteoblasts. Likewise, in a 10-year follow-up of PLIF, PRP combined with local bone graft increased the bone fusion area without any significant adverse response [222]. However, a systemic review and meta-analysis concluded that PRP could not contribute significantly in bone fusion as well as pain relief [223]. Although PRP-containing growth factors possess the potential to induce bone formation and fusion, the lack of a continuous pattern limits reaching a clear consensus [215]. Consequently, combined strategies of PRP and MIS-TLIF need to be explored to establish its therapeutic potential.

## 5. Future Prospects and Conclusions

MIS-TLIF is becoming a popular choice for lumbar arthrodesis, and choices of materials for bone fusion are continuously improving spinal fusion. Although ICGB is considered as the most suitable option for graft materials, the difficulty in autologous harvest and risk of associated viral infection limits its broad applicability. Compared to ICBG, the development of ceramics is comparatively easier, which also reduces the risk of infection. However, a proportional increase in pore size may compromise the strength of the ceramic bioscaffold graft. Hence, the autograft and allograft ceramics are also being applied. The cell-based regenerative medicine including stem cells, cellular bone matrices (CBMs), and PDBs also holds immense potential to regenerate injured tissues during TLIF. Most of the cell-based regenerative studies have been conducted in PLIF rather than MIS-TLIF, yet indicate its potential success in MIS-TLIF. Similarly, pre-clinical and clinical studies have revealed the potential of PRP-contained osteogenic and osteoconductive growth factors in enhancing bone fusion rate; however, extensive studies are needed to clearly establish its role in spinal surgery. In particular, the dual therapy of stem cells with PRP may be explored to confirm the elevated efficacy and safety in TLIF surgery. Since 3D printing could assist in designing a complex internal geometry of interbody cages with desired roughness and porosity, it could be synergistically applied with stem cells and PRP to achieve elevated osteogenesis and bone fusion during MIS-TLIF. Conclusively, although MIS-TLIF has achieved considerable success in spinal surgery, it could be synergized with ceramics, 3D printing, and regenerative therapeutics to achieve better clinical outcomes.
